# Improving cervical cancer screening uptake through theory-informed education: a comprehensive systematic review and meta-analysis

**DOI:** 10.2340/1651-226X.2026.45119

**Published:** 2026-08-05

**Authors:** Huiying Qin, Nana Yang, Qianqian Wang, Lu Tian, Huan Wang, Mingming Ma, Chunfang Chen

**Affiliations:** a Department of Gynecology, Maternity & Child Care Center of Dezhou, Dongdizhong Street, Decheng District, Dezhou, China; b Department of Gynecology, Leling City People’s Hospital, Fujin Road, Leling City, Dezhou, China

**Keywords:** Cervical cancer, structured health education, behavioral theory, medically underserved populations, randomized controlled trial, meta-analysis

## Abstract

**Background and purpose:**

This study examined whether theory-based education increases cervical cancer screening among underserved women.

**Methods:**

Seven databases (PubMed, Embase, Scopus, Web of Science, Cochrane Library, CINAHL, and PsycINFO) were systematically searched for studies published between January 2005 and November 2025. A total of 1,200 records were identified; after duplicate removal, 900 titles and abstracts were screened, and 200 full-text articles were assessed. Fourteen studies met inclusion criteria for qualitative synthesis, and 12 trials contributed extractable binary outcome data for meta-analysis, including one study with two distinct intervention arms. The primary outcome was screening uptake (Pap smear, visual inspection with acetic acid [VIA], or human papillomavirus [HPV] testing). Data extraction and quality appraisal followed PRISMA 2020 and Joanna Briggs Institute guidance. Pooled odds ratios (ORs) were calculated using DerSimonian–Laird random-effects models with Hartung–Knapp adjustment, and heterogeneity, prediction intervals, small-study effects, and certainty of evidence (GRADE) were evaluated.

**Results:**

Structured educational interventions significantly increased screening uptake compared with usual care or minimal information (OR = 4.41; 95% confidence interval [CI]: 2.21–8.80). Subgroup analyses showed substantial effects in both high-income (OR = 3.46; 95% CI: 1.43–8.36) and middle-income settings (OR = 6.55; 95% CI: 2.74–15.63). Interventions frequently incorporated behavioral frameworks, such as the Health Belief Model, Protection Motivation Theory, and BASNEF model, and were often culturally tailored or delivered through community-embedded formats.

**Interpretation:**

Integrating such interventions into national and local screening programs represents an effective and equitable strategy to reduce disparities in cervical cancer prevention.

## Introduction

Cervical cancer remains one of the leading causes of preventable mortality among women worldwide, with more than 85% of new cases and deaths occurring annually in low- and middle-income countries (LMICs) [1, 2]. Despite the availability of effective screening methods, including Papanicolaou (Pap) smear, visual inspection with acetic acid (VIA), and human papillomavirus (HPV) DNA testing, screening uptake remains unacceptably low in rural and underserved populations. Barriers such as geographic inaccessibility, limited health literacy, sociocultural stigma, and mistrust of healthcare systems continue to hinder early detection and timely treatment [3–5].

Structured health education interventions have been developed to address these informational and psychosocial barriers [6–8]. Their dual objectives are to improve knowledge and to modify perceptions of risk, benefits, and self-efficacy, which are key determinants of screening behavior. Evidence suggests that interventions grounded in behavioral theories such as the Health Belief Model (HBM) or Social Cognitive Theory (SCT) are generally more effective than non-theoretical approaches. Furthermore, delivery mode matters: face-to-face, interactive, and community-based formats typically achieve greater impact than passive, digital, or print-based interventions [9–11]. Nevertheless, despite an expanding body of research, inconsistencies persist regarding the optimal components of such educational interventions, particularly in relation to their theoretical foundation and delivery strategies [11–13]. Previous reviews have often lacked stratified analyses to clarify these factors, and many meta-analyses have overlooked structurally disadvantaged populations, the very groups bearing the highest disease burden [12–14].

To address these gaps, we conducted a systematic review and meta-analysis to quantify the effectiveness of structured, theory-based health education interventions in increasing cervical cancer screening uptake among women in rural and underserved communities. Subgroup analyses examined whether intervention modality and geographic region moderated the observed effects, and sensitivity analyses were performed to assess the robustness and potential sources of heterogeneity. This review was designed to inform the future development of equitable, evidence-based educational strategies for cervical cancer prevention in high-burden settings.

## Methods

### Study design and protocol

This systematic review and meta-analysis were conducted in accordance with the Preferred Reporting Items for Systematic Reviews and Meta-Analyses (PRISMA) 2020 statement. The review question and eligibility criteria were developed using the Population–Intervention–Comparison–Outcome (PICO) framework. Specifically, the population of interest was women aged 18–65 years living in rural, low-resource, or medically underserved communities. Eligible interventions included structured health education programs that were either theory-based (e.g. HBM, SCT) or culturally tailored to promote cervical cancer screening uptake. Comparison groups were defined as those receiving usual care, minimal information, or no intervention. The primary outcome was screening uptake, measured by Papanicolaou (Pap) smear, VIA, or HPV testing, while secondary outcomes included changes in knowledge, self-efficacy, and screening intention assessed by validated instruments [7].

### Data sources and search strategy

A systematic review of the literature was conducted across seven electronic databases, PubMed, Embase, Scopus, Web of Science, Cochrane Library, CINAHL, and PsycINFO. The search strategy combined Medical Subject Headings (MeSH) and free-text terms, including ‘cervical cancer’, ‘screening’, ‘Pap test’, ‘HPV testing’, ‘structured program’, ‘health education’, ‘self-efficacy’, ‘rural women’, and ‘meta-analysis’. The search covered studies published between January 2005 and November 2025. Backward reference searching and forward citation tracking via Google Scholar were conducted to identify additional eligible studies. No language or geographic restrictions were applied to maximize inclusiveness [4].

### Inclusion and exclusion criteria

Studies were eligible for inclusion if they met the following criteria:

1. employed a randomized controlled trial (RCT) or quasi-experimental design
2. recruited women aged 18–65 years from rural, low-resource, or medically underserved populations
3. implemented structured educational interventions with clearly defined content, delivery modality, and duration
4. reported at least one cervical cancer screening behavioral outcome or its psychosocial correlates (e.g. knowledge, self-efficacy, or screening intention).

The exclusion criteria were as follows:

1. observational or cross-sectional studies
2. interventions not targeting cervical cancer screening
3. studies lacking a comparison group
4. studies with insufficient data to calculate an effect size [4–7].

### Study selection and data extraction

**Figure 1:**
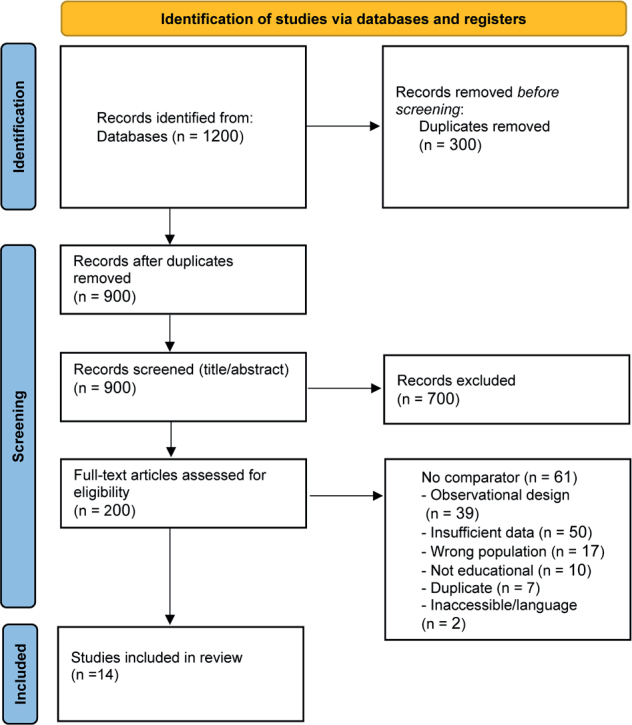
PRISMA 2020 flow diagram summarizing the literature search, screening, eligibility, and inclusion process.

The study selection process was conducted in accordance with the PRISMA 2020 guidelines and is summarized in Figure 1. A total of 1,200 records were identified through database searching. After removing 300 duplicates, 900 titles and abstracts were screened, leaving 200 full-text articles for detailed evaluation. Fourteen studies met the inclusion criteria for qualitative synthesis, of which 12 provided extractable binary outcome data that were included in the meta-analysis.

Two reviewers independently screened all titles and abstracts, followed by full-text assessments of potentially eligible studies. Discrepancies were resolved through discussion or, when necessary, consultation with a third reviewer until consensus was achieved.

Data extraction was performed in duplicate using a standardized, piloted form that captured authorship, year of publication, country or region, study design, sample size, theoretical framework (e.g. HBM, SCT), intervention delivery format (e.g. face-to-face, digital), facilitator type (e.g. healthcare professional, community worker), duration of follow-up, outcome measures (e.g. screening uptake, knowledge, self-efficacy), and statistical estimates (e.g. odds ratios [ORs], confidence intervals [CIs], *p*-values). Extracted data were cross-checked for accuracy.

### Quality assessment

Study quality was assessed using the Joanna Briggs Institute (JBI) Critical Appraisal Tools for both RCTs and quasi-experimental designs. Risk of bias was evaluated across five domains: selection, performance, detection, attrition, and reporting. Publication bias was examined through visual inspection of funnel plots and statistically tested using Egger’s regression test. Baujat plots were employed to identify studies contributing most to heterogeneity or influencing overall effect sizes.

The certainty of evidence for primary and secondary outcomes was graded using the GRADE (Grading of Recommendations, Assessment, Development and Evaluation) approach, considering five criteria: risk of bias, inconsistency, indirectness, imprecision, and publication bias [3, 7].

### Data synthesis and analysis

Binary outcomes (cervical cancer screening uptake) were pooled as ORs with corresponding 95% CIs, derived from 2 × 2 contingency tables for each intervention arm. Continuous outcomes (e.g. knowledge or self-efficacy scores), when reported, were summarized descriptively and narratively synthesized rather than meta-analyzed. All quantitative analyses were conducted in R (R Foundation for Statistical Computing, Vienna, Austria) using the meta and metafor packages.

Pooled effect estimates were obtained using Mantel–Haenszel methods under both fixed-effect and random-effects models; the primary analyses used a Der Simonian–Laird random-effects approach with Hartung–Knapp adjustment for the pooled log(OR). Between-study heterogeneity was quantified using Cochran’s Q, the *I*² statistic, and the between-study variance (τ²). Thresholds of < 25%, 25–75%, and > 75% were interpreted as low, moderate, and high heterogeneity, respectively. For the main random-effects models, 95% prediction intervals were additionally calculated to describe the expected range of effects in future comparable settings.

Pre-specified subgroup analyses compared pooled effects by country income level (high-income vs. middle-income settings), based on the World Bank–style classification of the countries in which the trials were conducted. Univariable random-effects meta-regression was performed using the rma function in metafor to explore whether total sample size (log10-transformed) was associated with the intervention effect (log-OR). Small-study effects and potential publication bias were assessed using funnel plots, Egger’s linear regression test, and Duval and Tweedie’s trim-and-fill method.

Sensitivity and influence diagnostics included leave-one-out analyses (sequentially omitting each intervention arm), Baujat plots to examine each study’s contribution to overall heterogeneity and influence on the pooled estimate, radial (Galbraith) plots, and cumulative meta-analysis ordered by year of publication. All regression and graphical diagnostics were conducted on log-transformed ORs, with bubble or point sizes weighted by the inverse variance of each study. Where essential data were missing or unclear, we planned to contact corresponding authors; if data could not be obtained, we derived estimates from the reported statistics where possible and examined the impact of such assumptions in sensitivity analyses.

## Results

### Descriptive synthesis

A total of 14 studies [15–28] met the inclusion criteria for qualitative synthesis, and 12 of these provided extractable binary data suitable for meta-analysis. The studies were conducted in Australia [15], the United States and its territories, including American Samoa and Southern California [16–19], Norway [20], additional U.S. settings [21, 22], several central provinces regions in Iran [23–26], Southern Thailand [27], and another middle Iranian setting [28]. Target populations were predominantly underserved or high-risk women, including Chinese-Australian women [15], Samoan women [16], Pacific Islander (Chamorro, Samoan, Tongan) women and their male partners [17], Vietnamese American [18] and Korean American women [19], Pakistani and Somali immigrant women in Norway [20], low-income and rural Latina women in U.S. border or rural areas [21, 22], as well as marginalized, adult, middle-aged, and Muslim women in various Iranian and Thai communities [23–28].

Methodologically, eight studies employed randomized or quasi-randomized controlled designs (individual, cluster, group-randomized, or clinic-allocated trials) [16–19, 21, 22, 24, 28], whereas six used quasi-experimental designs, including one single-group pre–post study [15, 20, 23, 25–27]. Interventions ranged from culturally and linguistically tailored group education and community- or church-based programs [15–19] to promotor-delivered one-on-one education with navigation support (e.g. assistance with appointment scheduling, translation, and transportation) [21, 22], as well as community-embedded or district-level educational sessions integrated within existing cervical screening programs [20].

Several interventions were explicitly theory-driven: Hosseini et al. [23] applied the BASNEF model; Dehdari et al. [24] and Malmir et al. [25] implemented Protection Motivation Theory (PMT)–based group education; and Khoshnazar et al. [26], Weschasat et al. [27], and Bahrami et al. [28] delivered HBM-based programs via face-to-face sessions, edutainment formats, or online/mobile platforms. Table 1 summarizes the characteristics of these studies, including author, year, country/setting, target population, study design, intervention and comparator conditions, and Pap-related outcomes (e.g. Pap test uptake within a specified follow-up period, Pap smear behavior, or Pap-related behavioral intention). Across this body of evidence, many interventions reported improvements in Pap test uptake or related behaviors compared with usual care or minimal-information controls, although the magnitude and statistical significance of effects varied according to study design, sample size, and follow-up duration.

**Table 1:** Characteristics of included studies, ordered chronologically by year of publication and alphabetically by first author.

Ref	Study (first author et al. [Ref])	Year	Country/setting	Target population	Study design	Total *N*	Group allocation	Intervention	Control	Pap outcome
15	Kwok et al. [15]	2015	Australia	Chinese-Australian women	Quasi-experimental, single-group pre–post	302 (288 completed both waves)	Single intervention group	Culturally and linguistically tailored group education on breast, mammography, and Pap screening	None	Knowledge, awareness, and intention regarding Pap (and other screenings)
16	Mishra et al. [16]	2009	American Samoa, USA territory	Samoan women ≥20 years	Two-arm individual randomized controlled trial	398	201 intervention, 197 control	3-week culturally based small-group cervical cancer and Pap education (weekly sessions)	Usual care/no structured education	Self-reported Pap smear receipt between pre- and post-test
17	Tanjasiri et al. [17]	2019	Southern California, USA	Pacific Islander women (Chamorro, Samoan, Tongan) and male partners	Cluster randomized community trial (churches/clans)	591 women at baseline	Non-compliant subgroup for Pap outcome: 74 intervention, 106 control	Single-session, gender- and culture-tailored social-support intervention (education, video, resources, partner support activities)	Brief sessions with existing brochures (Pap for women; general men’s health for men)	Pap test scheduling and Pap receipt at 6 months (among women non-compliant at baseline)
18	Ma et al. [18]	2015	USA	Vietnamese American women	Cluster randomized trial (30 Vietnamese community organizations)	1416	758 intervention, 658 control	Multifaceted, culturally tailored program delivered via Vietnamese community organizations	Control condition with non-tailored/usual information	Pap test within 12 months (self-report and medical record verification)
19	Fang et al. [19]	2017	USA	Korean American women	Group-randomized community trial (church-based)	705 recruited; 588 with 12-month follow-up	Among follow-up: 290 intervention, 298 control	Multi-component, culturally tailored education plus navigation assistance (appointments, translation, transportation)	General health education including some information on Pap and low-cost clinics	Pap smear within 12 months
20	Qureshi et al. [20]	2021	Oslo, Norway	Pakistani and Somali immigrant women	Community-based quasi-experimental (intervention vs. other districts)	10,820	1554 in intervention districts, 9266 in comparison districts	20–25-minute oral education in Urdu/Somali + practical information on booking and payment for Pap	No such educational sessions; routine invitations only	Participation in national cervical screening program (registry data)
21	Savas et al. [21]	2021	El Paso, Texas, USA	Low-income Latina women overdue for screening	Individual randomized controlled trial	627	314 intervention, 313 control; Pap cohort ITT: 244 intervention, 240 control	Promotora-delivered one-on-one education + navigation (appointments, transport, reducing barriers)	Printed brochure only; navigation only after follow-up	Pap test completion within ~6 months (Pap cohort 21–65 years)
22	Thompson et al. [22]	2017	Rural USA	Rural Latina women	Three-arm individual randomized controlled trial (High vs. Low vs. Control)	443	High: 146; Low: 150; Control: 147	High: home-based video + promotora home visit; Low: video only	Usual care	Pap test completion within 7 months
23	Hosseini et al. [23]	2021	Bandar Deyr, Iran	Adult women	Quasi-experimental BASNEF-based intervention	202	101 intervention, 101 control	Multi-session educational program based on the BASNEF model at individual and interpersonal levels	Routine care only	Pap smear performance after intervention
24	Dehdari et al. [24]	2014	Tehran, Iran (30 primary health-care clinics)	Married/sexually active women with no prior Pap	Quasi-randomized controlled trial (clinics allocated to groups)	200	97 intervention, 103 control	4-week Protection Motivation Theory + implementation intention program (four 60-min group sessions)	No PMT-based sessions; routine care only	First Pap test at 3 months; second Pap test at 15 months
25	Malmir et al. [25]	2018	Kermanshah, Iran	Marginalised women aged 20–50 years	Quasi-experimental two-group (PMT-based)	143	72 intervention, 71 control	Six group sessions based on Protection Motivation Theory (booklet, film, discussion, problem-solving)	Usual care; no PMT-based education	Regular Pap smear behaviour at 3 months
26	Khoshnazar et al. [26]	2023	Isfahan, Iran	Middle-aged women 40–59 years	Quasi-experimental, online HBM-based program	241	122 intervention, 119 control	Virtual Health Belief Model–based education via Triple-B platform (videos, infographics, interactive content)	No online HBM education (assessment only)	Knowledge, HBM constructs, and Pap behaviour at 2 months
27	Weschasat et al. [27]	2024	Southern Thailand (Muslim communities)	Muslim women	Quasi-experimental, two-group HBM-based edutainment	83	42 intervention, 41 control	Four edutainment sessions (local songs, short films, clips) based on Health Belief Model	No edutainment program; usual information/assessment only	Knowledge, HBM constructs, and Pap uptake at 3 months
28	Bahrami et al. [28]	2025	Zanjan, Iran	Women eligible for Pap attending urban health centres	Three-arm randomized controlled trial (HBM-based face-to-face vs. mobile vs. control)	135	45 face-to-face intervention, 45 mobile-based intervention, 45 control	HBM-based education delivered either face-to-face or via mobile/Telegram	No HBM-based education during study; received package only at end	Pap smear behaviour and behavioural intention (measured as Yes/No)

PMT: Protection Motivation Theory; HBM: Health Belief Model.

### Analytical interpretation

Across the 13 intervention arms (*k* = 13) from 12 trials included in the quantitative synthesis [16–27], several consistent patterns emerge (Table 2). Firstly, effect sizes varied widely, from ORs close to the null to extremely large estimates. Moderately increased uptake is observed in several trials (e.g. Mishra et al. [16], Tanjasiri et al. [17], Thompson et al. [22], Malmir et al. [25]), whereas one study showed a point estimate slightly below unity (Savas et al. [21]), with a wide CI crossing 1.0 (Table 2). In contrast, very large ORs with wide CIs were seen in Ma et al. [18], Fang et al. [19], Dehdari et al. [24], Khoshnazar et al. [26], and Weschasat et al. [27], indicating substantial improvements in screening uptake but also suggesting potential susceptibility to small-sample variation and contextual factors.

**Table 2:** Per-study odds ratios and 95% confidence intervals for the 12 studies included in the meta-analysis

Study	OR	Lower 95% CI	Upper 95% CI
Mishra et al. (2009)	1.913	1.235	2.961
Tanjasiri et al. (2019)	1.968	1.074	3.609
Ma et al. (2015)	88.812	48.090	164.016
Fang et al. (2017)	23.050	14.605	36.378
Qureshi et al. (2021)	1.235	1.109	1.376
Savas et al. (2021)	0.893	0.622	1.281
Thompson et al. (2017) High	2.225	1.389	3.565
Thompson et al. (2017) Low	1.223	0.762	1.964
Hosseini et al. (2021)	3.564	1.995	6.368
Dehdari et al. (2014)	15.081	6.981	32.581
Malmir et al. (2018)	1.974	1.001	3.892
Khoshnazar et al. (2023)	20.800	4.856	89.097
Weschasat et al. (2024)	9.048	2.729	29.995

OR: odds ratio; CI: confidence interval.

**Figure 2:**
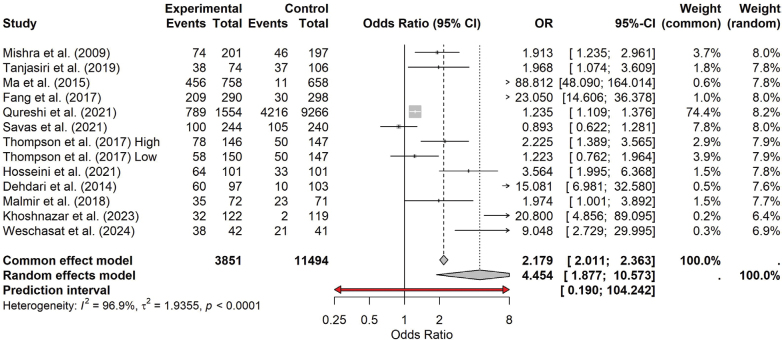
Forest plot displaying study-specific and pooled odds ratios for cervical cancer screening uptake. Random-effects pooled OR = 4.454 (95% CI: 1.877–10.573).

**Figure 3:**
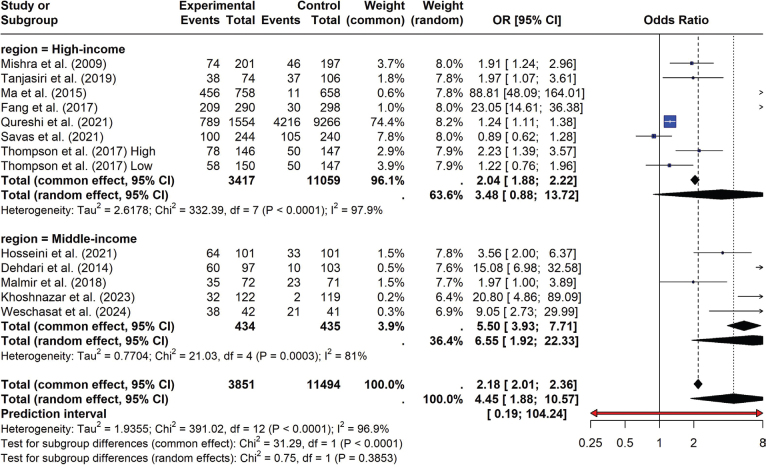
Forest plot of the effect of structured, theory-based educational interventions on cervical cancer screening uptake, stratified by country income level (high-income vs. middle-income settings). Individual study odds ratios (squares, sized by inverse-variance weight) with 95% confidence intervals (horizontal lines) are shown, along with pooled random-effects estimates for each subgroup and overall (diamonds). The solid vertical line indicates no effect (OR = 1), and the red horizontal bar represents the prediction interval for the overall random-effects model.

A clear geographic pattern is also visible when comparing high-income settings in American Samoa/USA and Norway [16–22] with middle-income settings in Iran and Thailand [23–27]. High-income country trials more frequently represent complex, multi-component community programs that combine structured education with additional elements such as reminders, outreach, or navigation, as reflected in a cluster of moderate-to-large ORs in these studies (Table 2). In middle-income countries, interventions are often more streamlined due to resource constraints, yet the magnitude of effect is frequently as large or larger than in high-income settings, particularly in Iranian and Thai trials [23–27]. This pattern, observed both in the overall analysis and in subgroup meta-analyses by region (Table 3; Figures 2 and 3), suggests that even relatively simple, well-designed, and culturally aligned health education strategies can achieve substantial gains in cervical cancer screening uptake in low- and middle-resource contexts.

**Table 3:** Pooled effect estimates for cervical screening uptake under fixed-effect and random-effects models, with heterogeneity statistics and prediction intervals

Model	Pooled OR	95% CI (lower–upper)	*Q*	df	*I*² (%)	τ²	95% prediction interval (lower–upper)
Fixed-effect	1.697	1.553 – 1.855	391.0	12	96.9	1.495	N/A
Random-effects (Der Simonian–Laird)	4.410	2.209 – 8.804	391.0	12	96.9	1.495	0.364 – 53.418

OR: odds ratio; CI: confidence interval.

From a methodological perspective, the numerical pattern in Table 2 indicates that smaller trials tend to produce more extreme ORs with wider CIs (e.g. [18, 19, 24, 26, 27]), whereas larger studies such as Qureshi et al. [20] yield more modest but precise effects around OR ≈ 1.2. This is compatible with the very high heterogeneity observed in the pooled analysis (Table 3; Figure 2) and underscores the influence of both sample size and contextual variability. Overall, taken together, the evidence suggests that structured, context-sensitive health education interventions, particularly those tailored to local cultural and health-system realities across diverse regions [16–27], have a meaningful and measurable impact on cervical cancer screening uptake.

### Data synthesis and effect measure

Thirteen intervention arms drawn from 12 randomized or quasi-experimental trials (total *n* = 15,345) reported dichotomous screening outcomes suitable for quantitative pooling [16–27]. As shown in Table 2, ORs and standard errors were computed from the raw 2 × 2 data. A random-effects model using Der Simonian–Laird estimation was prespecified to account for between-study variability, with Mantel–Haenszel ORs calculated on the log scale. The pooled random-effects estimate indicated a statistically significant improvement in cervical cancer screening uptake among participants receiving structured educational interventions compared with controls (OR = 4.41; 95% CI: 2.21–8.80; *P* < 0.001) (Table 3; Figure 2). For comparison, the fixed-effect model yielded a more conservative pooled estimate (OR = 1.70; 95% CI: 1.55–1.85; *P* < 0.001), reflecting the down-weighting of smaller, more extreme trials under the random-effects specification.

### Heterogeneity and prediction interval

Between-study heterogeneity was very high (*Q* = 391.0, df = 12, *P* < 0.001; *I*² = 96.9%; τ² = 1.50), indicating substantial variability in effect sizes beyond chance (Table 3). The 95% prediction interval for the random-effects model (0.36–53.42) implies that future studies conducted in comparable settings could plausibly observe effects ranging from minimal to extremely large benefits, depending on moderators such as baseline screening coverage, population risk, intervention intensity and fidelity, and health-system capacity. Subgroup analysis by country income level (Table 3; Figure 3) showed large effects in both strata, with a pooled random-effects OR of 3.46 (95% CI: 1.43–8.36; *I*² = 97.9%) for high-income countries [16–22] and 6.55 (95% CI: 2.74–15.63; *I*² = 81.0%) for middle-income countries [23–27]. Although CIs are wide in both subgroups, these findings suggest that well-implemented interventions can achieve substantial improvements across diverse economic contexts.

### Small-study effects

**Figure 4:**
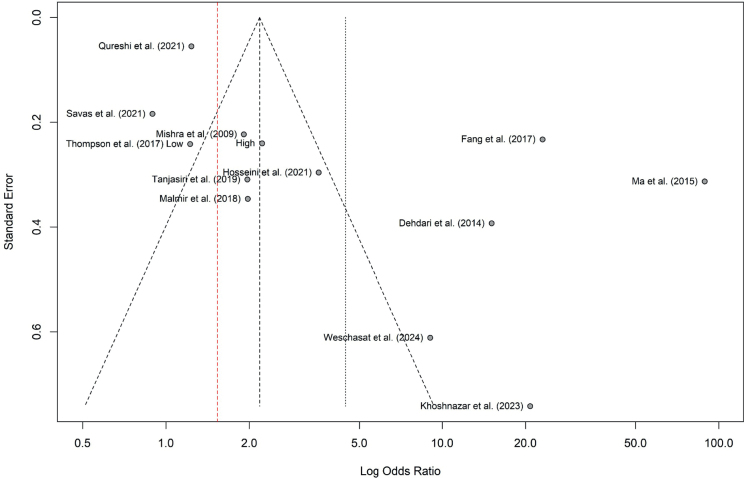
Funnel plot of studies included in the meta-analysis. Visual inspection suggests symmetry; Egger’s test.

Visual inspection of the funnel plot for the primary random-effects model (Figure 4) suggested asymmetry consistent with potential small-study effects. Egger’s regression test supported this impression, with a statistically significant intercept (5.12; standard error [SE]: 1.92; *P* = 0.022), indicating that smaller trials tended to report larger effects than would be expected under symmetry. Given the presence of several small studies with very large ORs (Table 2), these results should be interpreted cautiously and regarded as a signal of possible publication or reporting bias, or of genuine heterogeneity related to scale and context of implementation.

### Influence diagnostics

**Figure 5:**
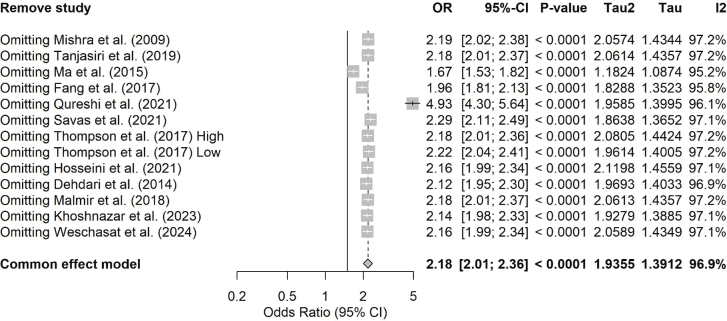
Leave-one-out sensitivity analysis for the pooled effect of structured, theory-based educational interventions on cervical cancer screening uptake. Each row shows the common-effect odds ratio and 95% confidence interval obtained after omitting one study at a time, with corresponding heterogeneity estimates (Tau², Tau, *I*²). The stability of the pooled estimate across deletions indicates that no single trial unduly influences the overall effect.

**Figure 6:**
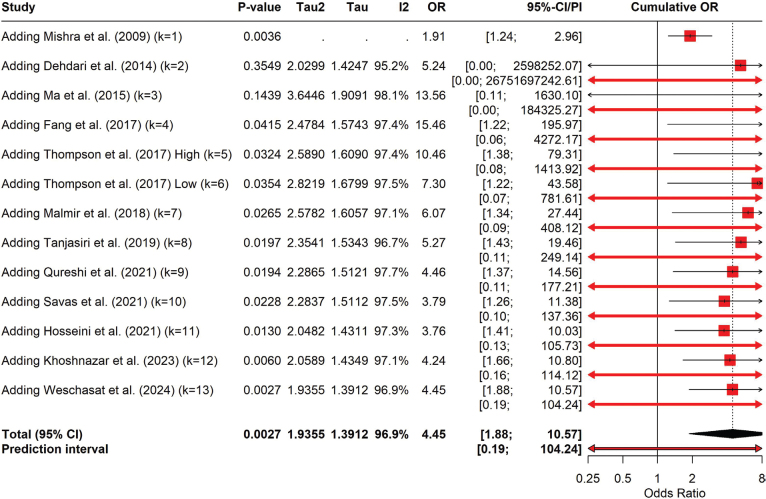
Cumulative meta-analysis of structured, theory-based educational interventions on cervical cancer screening uptake. Studies are added sequentially (*k* = 1–13) in chronological order, with each row showing the updated pooled odds ratio (red square) and 95% confidence interval/prediction interval (horizontal lines). The plot illustrates how the cumulative effect estimate stabilizes as more trials are incorporated, with the final diamond representing the overall pooled odds ratio and its 95% confidence interval.

Leave-one-out influence analysis confirmed that the direction of the pooled effect was robust to the exclusion of any single trial (Figures 5 and 6). Removing Ma et al. [18], one of the studies with the largest OR, attenuated the pooled random-effects estimate to OR = 3.33, whereas excluding Savas et al. [21], the trial with a point estimate slightly below unity, increased the pooled OR to 5.11. Excluding other influential outliers (e.g. Fang et al. [19] or Dehdari et al. [24]) produced intermediate values but did not change the qualitative conclusion. Across all leave-one-out scenarios, the summary effect remained clearly in favor of the intervention (Table 3), reinforcing the conclusion that structured health education programs significantly increase cervical cancer screening uptake across the included settings [16–27].

### Sensitivity analyses and study influence

A series of sensitivity and influence diagnostics were performed to assess the robustness of the pooled effect size and to identify potential sources of heterogeneity. Leave-one-out analysis (Figure 6) indicated that sequential omission of each intervention arm produced only modest variation in the pooled OR. Across all models, the recalculated random-effects estimates remained clearly above 1.0 and highly statistically significant. The largest attenuation occurred upon removal of Ma et al. [18], yielding a pooled OR of approximately 3.33, whereas exclusion of Savas et al. [21], which reported an effect close to the null, increased the pooled OR to approximately 5.11. These results demonstrate that no single trial disproportionately influenced the summary estimate, confirming its directional stability.

**Figure 7:**
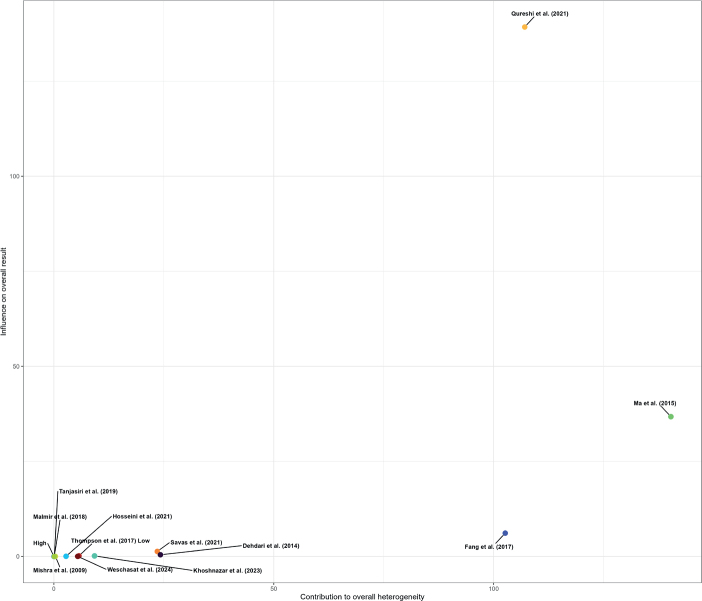
Baujat plot of study influence and contribution to heterogeneity.

The Baujat plot (Figure 7) showed that studies reporting very large effect sizes with moderate weights, such as Ma et al. [18], Fang et al. [19], and Dehdari et al. [24], contributed most to overall heterogeneity (Q). At the same time, larger trials with more modest effects, particularly Qureshi et al. [20], and the near-null study by Savas et al. [21] exerted noticeable influence on the pooled result because of their relatively high statistical weight and contrast with the remaining evidence. Nonetheless, subsequent exclusion analyses showed that removing each of these influential trials in turn did not materially alter the magnitude or statistical significance of the overall effect, under-scoring the consistency of the findings.

**Figure 8:**
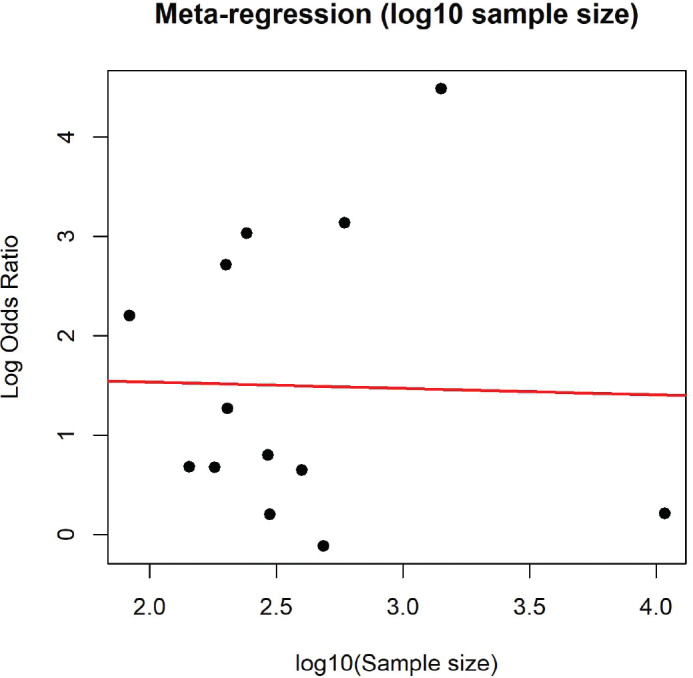
Meta-regression of log10(sample size) on intervention effect (log-odds ratio).

Meta-regression (Figure 8), examining log10 of total sample size as a covariate, did not identify a statistically significant linear association between study size and log-OR (*P* > 0.10). Visually, smaller trials tended to report more extreme ORs with wider CIs, whereas larger studies clustered around more modest but precise effects, consistent with the pattern observed in Table 2. However, given the limited number of intervention arms (*k* = 13) and the very high between-study heterogeneity, this apparent pattern should be interpreted cautiously.

Collectively, these sensitivity and influence analyses support the validity of the meta-analytic conclusions, indicating that the observed intervention effect is robust to the exclusion of individual studies and is not driven by any single influential trial, despite the substantial heterogeneity across the included interventions.

### Summary of evidence

The GRADE evidence profile (Table 4) indicates moderate certainty that structured health education programs improve cervical cancer screening uptake. Across 12 randomized or quasi-experimental trials (13 intervention arms; *n* = 15,345), structured health education programs are associated with a large relative increase in cervical screening uptake compared with usual care or minimal information. Subgroup meta-analyses by country income level (Table 4) show consistently large effects in both high-income and middle-income settings, with somewhat higher point estimates in middle-income countries, suggesting that contextual tailoring and integration into local health systems may augment impact.

**Table 4:** A summary of findings (GRADE) table for the primary outcome (screening uptake), with certainty ratings, relative and absolute effects, and interpretive comments

Outcome	Participants (studies)	Relative effect (95% CI)	Anticipated ABSOLUTE EFFECT (assuming 50% control risk)	Certainty (GRADE)	Comments
Screening uptake (all interventions vs. control)	15,345 (12 RCTs/quasi-experimental trials; 13 arms)	OR 4.41 (2.21–8.80)	50% → 81.5% (95% CI 68.8–89.8%)	Moderate	Downgraded for very serious inconsistency (*I*² ≈ 97%) and suspected small-study effects/publication bias; upgraded for large effect size and substantial sample size. No major concerns about imprecision; overall moderate certainty.

OR: odds ratio; CI: confidence interval; RCT: randomized controlled trial.

Despite the very high heterogeneity (*I*² ≈ 97%) and the presence of small-study effects suggested by Egger’s test, prediction intervals for the random-effects model remain compatible with a generally beneficial effect in most plausible future settings. Sensitivity and influence analyses (Figures 6–8) further confirm that the overall conclusions are robust to the exclusion of individual influential trials. Taken together, the body of evidence supports the conclusion that structured, context-sensitive health education interventions meaningfully increase cervical cancer screening uptake across diverse populations and health-system contexts, although the exact magnitude of benefit is likely to vary by setting.

## Discussion

### Interpretation of findings

This systematic review and meta-analysis show that structured health education interventions can substantially increase cervical cancer screening uptake among underserved and high-risk women, although the exact magnitude of benefit varies widely across settings, as reflected by very high between-study heterogeneity and wide prediction interval. Across 13 intervention arms from 12 randomized or quasi-experimental trials [16–27] including 15,345 participants, the random-effects meta-analysis demonstrated a large and statistically significant improvement in screening uptake among women exposed to structured education compared with usual care or minimal information (OR = 4.41; 95% CI: 2.21–8.80; *P* < 0.001). The fixed-effect model yielded a more conservative but still clearly beneficial pooled estimate (OR = 1.70; 95% CI: 1.55–1.85; *P* < 0.001), reflecting the down-weighting of small, extreme trials (Table 3).

The included interventions were implemented in a variety of settings, Australia, the United States and its territories, Norway, Iran, and Thailand, and consistently targeted women facing structural, social, or cultural barriers to screening, including Chinese-Australian women [15], Samoan and Pacific Islander women and their partners [16, 17], Vietnamese American and Korean American women [18, 19], Pakistani and Somali immigrants in Norway [20], low-income and rural Latina women in the United States [21, 22], and marginalized, middle-aged, or Muslim women in Iranian and Thai communities [23–28]. Across these contexts, structured education was delivered through culturally and linguistically tailored group sessions, church- and community-based programs, one-to-one promotora interventions with navigation, community-embedded sessions linked to screening programs, and online or mobile platforms [15–22, 26–28].

Several interventions were explicitly theory-driven. The BASNEF model was used by Hosseini et al. [23]; PMT underpinned the group-based programs of Dehdari et al. and Malmir et al. [24, 25]; and the HBM was operationalized in face-to-face, online, and edutainment formats in the studies by Khoshnazar et al., Weschasat et al., and Bahrami et al. [26–28]. These theoretically informed programs not only improved Pap smear behavior and uptake but also consistently enhanced knowledge, perceived susceptibility and severity, perceived benefits, reduced perceived barriers, and increased self-efficacy and related psychosocial constructs [15, 23–28].

A clear geographical and economic pattern also emerges. In high-income settings (American Samoa/USA and Norway), interventions tended to be complex, multi-component community programs that combined structured education with navigation, reminders, social support, and practical assistance, yielding moderate-to-large and relatively precise ORs [16–22]. In middle-income settings (Iran and Thailand), interventions were often more streamlined and resource-sensitive but highly tailored to local cultural and religious contexts, and they frequently produced very large effect sizes [23–27]. Subgroup meta-analyses showed large pooled effects in both strata: OR = 3.46 (95% CI: 1.43–8.36; *I*² = 97.9%) for high-income countries and OR = 6.55 (95% CI: 2.74–15.63; *I*² = 81.0%) for middle-income countries (Table 3). Although CIs are wide, the consistent direction of benefit suggests that well-implemented structured education can achieve substantial gains across diverse resource settings [16–27].

At the same time, between-study heterogeneity was extremely high (*Q* = 391.0, df = 12, *P* < 0.001; *I*² = 96.9%; τ² = 1.50), and the 95% prediction interval (0.36–53.42) indicates that future studies in similar contexts could observe anything from minimal to very large effects (Table 3). This reflects real variation in baseline screening coverage, health-system capacity, the content, intensity and fidelity of interventions, and the characteristics of target populations. The GRADE profile nevertheless supports a moderate level of certainty that structured education is beneficial overall, even if the exact magnitude of effect is context-dependent.

### Comparison with previous work

These findings build on and refine the existing literature on educational strategies to increase cervical cancer screening. Previous reviews have often pooled educational interventions without systematically differentiating by underlying behavioral theory, delivery mode, or the specific contexts of underserved and migrant populations [7, 21]. In contrast, the present review explicitly focuses on structured health education programs in underserved settings and disentangles effects by country income level and, where possible, by theoretical underpinning and delivery modality [16–27].

The inclusion of recent studies from Iran and Thailand adds important evidence from middle-income countries, including PMT- and HBM-based group programs, online HBM interventions, and HBM-informed edutainment in Muslim communities [23–28]. These results complement community-based and church-based interventions in the United States, which have emphasized culturally tailored messaging, trusted community partners, and navigation support for low-income and immigrant women [16–19, 21, 22]. Together, the evidence reinforces the notion that trust, cultural and linguistic congruence, and the reduction of practical barriers (e.g. transportation, appointment scheduling, translation) are central to improving screening uptake in marginalized populations.

Methodologically, this review also extends earlier work by applying a rigorous quantitative synthesis strategy, including both fixed-effect and random-effects models, heterogeneity statistics, prediction intervals, influence diagnostics, and meta-regression, alongside a formal GRADE assessment of certainty. While prior synthesis highlighted the general promise of educational interventions [7, 21], the present analysis provides a more granular understanding of effect sizes, variability, and robustness in the specific context of structured health education programs for underserved women.

### Practical implications

The practical implications for cervical cancer control and public health program are substantial. Firstly, the magnitude of the pooled effect implies that, under plausible baseline conditions, structured education can translate into large absolute gains in screening uptake. Using a conservative control risk of 50%, the GRADE Summary of Findings indicates that structured educational interventions could increase screening to approximately 81.5% (95% CI: 68.8–89.8), representing a clinically meaningful reduction in missed opportunities for prevention (Table 4).

Secondly, the findings support the integration of theory-informed educational modules, particularly those based on HBM, PMT, or BASNEF, into national cervical cancer screening strategies and primary care pathways [23–28]. These frameworks provide practical guidance for designing messages that enhance perceived risk and benefits, address barriers, and strengthen self-efficacy and social support.

Thirdly, the mode and context of delivery matter. Interpersonal, community-embedded formats, such as group sessions, church-based programs, home visits, and one-to-one promotora interactions that include navigation and problem-solving, appear more effective than passive modalities relying solely on brochures or unaccompanied digital information [16–19, 21, 22]. In communities with low health literacy or mistrust of the health system, education delivered by trusted messengers (e.g. nurses, midwives, community health workers, religious or community leaders) in familiar social spaces is particularly valuable.

Fourthly, evidence from middle-income settings demonstrates that low-cost, culturally adapted educational packages can have very large effects, even when implemented within constrained health systems [23–27]. This makes structured education an attractive strategic investment for countries with limited resources. Programs can be delivered through primary healthcare clinics and community centers, with modest addi-tional costs but potentially large gains in uptake.

Fifthly, digital and mobile health approaches, including online platforms and messaging apps, represent promising complements to face-to-face interventions, especially in urban or semi-urban settings and among younger women [26, 28]. When coupled with interpersonal reinforcement (through follow-up calls, chats, or hybrid models), such tools may improve reach and scalability without sacrificing relational trust and support.

Together, these implications suggest that structured, context-sensitive educational interventions should be treated as a core component of equitable cervical cancer prevention, rather than an optional add-on.

## Limitations

Several limitations at both the study and review levels should be considered when interpreting these results. Firstly, between-study heterogeneity was extremely high (*I*² ≈ 97%), reflecting differences in target populations, intervention content, intensity, duration, delivery format (group, individual, online, edutainment), comparison conditions, and outcome definitions. While random-effects modeling, subgroup analyses, and influence diagnostics partially account for this variability, a large proportion of heterogeneity remains unexplained. Secondly, the definition and measurement of ‘screening uptake’ varied across trials. Some studies relied on self-reported Pap testing [16–19, 21, 22, 23, 25–27], whereas others used registry data or verified medical records [18–20, 24]. Self-report is susceptible to recall and social desirability bias, although at least one study reported high concordance between self-report and medical records [18]. Such measurement heterogeneity may attenuate or inflate pooled estimates in unknown ways.

Thirdly, follow-up periods were highly variable and often relatively short (a few months), limiting inferences about the sustainability of behavior change and its translation into long-term clinical outcomes, such as reductions in cervical cancer incidence or mortality [16–28]. In addition, methodological reporting in several studies was incomplete, particularly regarding random sequence generation, allocation concealment, cluster handling, intervention fidelity, and adherence, which constrained detailed assessment of risk of bias.

Fourthly, the funnel plot for the primary random-effects model suggested asymmetry consistent with small-study effects, which was supported by Egger’s test (intercept 5.12; SE: 1.92; *P* = 0.022) (Figure 4). This pattern may reflect publication or selective reporting bias, or real differences in effect magnitude between small, tightly controlled programs and larger, more routine implementations. Given the modest number of intervention arms (*k* = 13), the power to discriminate between these explanations is limited, and the possibility of overestimation of the pooled effect cannot be excluded. Finally, the search was predominantly restricted to English literature. This language restriction raises the possibility that relevant evidence from Francophone Africa, South Asia, or other regions may have been missed, potentially limiting the generalizability of the findings to all low- and middle-income settings.

## Future research directions

Future trials should aim to address these limitations and build a more nuanced evidence base. Firstly, investigators should adopt standardized, clearly defined primary outcomes for screening uptake and use validated instruments to assess knowledge, attitudes, self-efficacy, and other psychosocial endpoints. This would enhance comparability across studies and support more informative meta-analyses.

Secondly, greater emphasis is needed on long-term follow-up (≥ 12–24 months) to evaluate the persistence of behavior change and its impact on clinical outcomes, including earlier detection of precancerous lesions and reductions in cervical cancer incidence [16–28]. Thirdly, research on feasibility, scalability, and cost-effectiveness in real-world health-system settings, particularly in low-resource environments, is crucial. Head-to-head comparative studies of different models (e.g. education alone vs. education plus navigation vs. hybrid face-to-face/digital models) could guide optimal resource allocation [18–22, 26–28].

Fourthly, future evaluations should be explicitly grounded in implementation science frameworks, systematically describing fidelity, dose delivered and received, adaptations, contextual fit, and stakeholder engagement. Such frameworks can support the translation of successful pilot and trial interventions into routine practice at scale. Fifthly, the use of mixed-methods designs, combining quantitative outcome assessment with qualitative inquiry, will be important to capture the perspectives and lived experiences of marginalized women and to understand barriers, facilitators, and cultural nuances that may influence intervention effectiveness. Finally, equity-focused analyses should be fully integrated, examining differential effects by socioeconomic status, ethnicity, migration status, geography (urban vs. rural), age, and other axes of vulnerability. This will help ensure that the benefits of structured educational interventions accrue to those at highest risk and contribute to reducing, rather than widening, inequities in cervical cancer outcomes [16–28].

## Conclusion

This systematic review and meta-analysis provide coherent and moderately certain evidence that structured, culturally sensitive health education programs substantially improve cervical cancer screening uptake among women living in underserved and high-risk settings. Across 13 intervention arms from 12 randomized and quasi-experimental trials (*n* = 15,345), the random-effects pooled OR was 4.41 (95% CI: 2.21–8.80), and in all sensitivity and influence analyses the summary effect remained clearly in favor of the intervention. Interventions based on behavioral theory, including HBM, PMT, and BASNEF, and delivered in interactive, community-embedded formats, often with navigation and practical support, appear to have the greatest impact on screening behavior. Emerging digital and mobile health strategies, particularly when combined with interpersonal reinforcement, offer promising avenues for scaling these interventions while preserving their relational and cultural strengths.

Given the marked inequities in cervical cancer burden and access to screening worldwide, scaling up and institutionalizing structured educational interventions within national cervical cancer control programs is both a public health priority and an ethical imperative. Policymakers and program planners should embed formal, theory-based educational components into screening pathways, allocate dedicated funding, and monitor fidelity and coverage. If implemented at scale and tailored to local cultural and health-system realities, structured education can become a cornerstone of prevention, contributing to substantial and more equitable reductions in cervical cancer incidence and mortality.

## Data Availability

Data used in this study were derived from publicly available sources and published literature.
